# Resolution among major placental mammal interordinal relationships with genome data imply that speciation influenced their earliest radiations

**DOI:** 10.1186/1471-2148-8-162

**Published:** 2008-05-27

**Authors:** Björn M Hallström, Axel Janke

**Affiliations:** 1 Department of Cell and Organism Biology, Division of Evolutionary Molecular Systematics, University of Lund, Sölvegatan 29, S-223 62 Lund, Sweden

## Abstract

**Background:**

A number of the deeper divergences in the placental mammal tree are still inconclusively resolved despite extensive phylogenomic analyses. A recent analysis of 200 kbp of protein coding sequences yielded only limited support for the relationships among Laurasiatheria (cow, dog, bat and shrew), probably because the divergences occurred only within a few million years from each other. It is generally expected that increasing the amount of data and improving the taxon sampling enhance the resolution of narrow divergences. Therefore these and other difficult splits were examined by phylogenomic analysis of the hitherto largest sequence alignment. The increasingly complete genome data of placental mammals also allowed developing a novel and stringent data search method.

**Results:**

The rigorous data handling, recursive BLAST, successfully removed the sequences from gene families, including those from well-known families hemoglobin, olfactory, myosin and HOX genes, thus avoiding alignment of possibly paralogous sequences. The current phylogenomic analysis of 3,012 genes (2,844,615 nucleotides) from a total of 22 species yielded statistically significant support for most relationships. While some major clades were confirmed using genomic sequence data, the placement of the treeshrew, bat and the relationship between Boreoeutheria, Xenarthra and Afrotheria remained problematic to resolve despite the size of the alignment. Phylogenomic analysis of divergence times dated the basal placental mammal splits at 95–100 million years ago. Many of the following divergences occurred only a few (2–4) million years later. Relationships with narrow divergence time intervals received unexpectedly limited support even from the phylogenomic analyses.

**Conclusion:**

The narrow temporal window within which some placental divergences took place suggests that inconsistencies and limited resolution of the mammalian tree may have their natural explanation in speciation processes such as lineage sorting, introgression from species hybridization or hybrid speciation. These processes obscure phylogenetic analysis, making some parts of the tree difficult to resolve even with genome data.

## Background

Recently the resolution of the mammalian tree made a quantum leap forward with the analysis of protein coding sequences of whole genome data [1-4]. The genome data allowed collecting the protein coding sequences from some 3000 genes, which is equivalent to 2.2 million nucleotides (Mnt), representing ≈10% of all coding sequences [2]. During the last 15 years since the influential review of Novacek [5] on the mammalian evolution, many previously uncertain relationships are now becoming consistently resolved by different data sets and analytical approaches. However, lately released mammal genome data have not been investigated by phylogenomic analyses.

Presently the monophyly of each of the four major clades, Euarchontoglires, Laurasiatheria, Xenarthra, and Afrotheria is supported by most analyses based on sequence data [2,6] and genome level data such as LINE and LTR elements [7]. The smallest clade, Xenarthra, consists of only two orders, Cingulata (armadillos) and Pilosa (sloths and anteaters) [8]. The Afrotheria [9] comprises the traditional orders Sirenia, Proboscidea, Hyracoidea, and Tubulidentata plus the members from the paraphyletic Lipotyphla (Insectivora): the Tenrecidae, Macroscelidea and Chrysochloridea. The Afrotheria and Xenarthra are grouped in the clade named Xenafrotheria [10], a clade that is supported by phylogenomic analyses [2] and retroposon and indel analysis [11]. Other studies, however, find support for a basal Xenarthra clade from retroposon data [7], or a basal Afrotheria clade from some sequence data analyses [3,12]. The two remaining clades, Euarchontoglires (Primates, Rodentia, Lagomorpha, Scandentia, Dermoptera) and Laurasiatheria (all remaining orders) together comprise the species rich taxon Boreoplacentalia, which is supported by all current analyses. The new name Boreoplacentalia has been suggested for this clade for being more consistent and specific than the previous name Boreoeutheria [13].

Many branches of the mammalian tree remained difficult to resolve, even with the analysis of several 100 thousand nt (knt) of sequence data. The most basal placental mammal divergences were inconclusive even with the analysis of some 200 knt of coding sequence data [3]. The analysis of a ten-fold larger dataset, 2.2 Mnt of protein coding sequence data supported the grouping of Xenarthra with the Afrotheria in the higher order clade Xenafrotheria [2]. Jackknife analysis showed that at least 600 knt sites of protein coding data were needed to significantly resolve this relationship [2]. The notoriously difficult to resolve split between Xenafrotheria and Boreoplacentalia took place at ≈100 million years ago (Mya). However, the two clades diverged into Xenarthra and Afrotheria, and Laurasiatheria and Euarchontoglires respectively only 3–4 million years (Myr) later [2]. The resolution of these narrow temporal occurrences required more data than available to the study of Nikolaev et al. [3], however the placement of Xenarthra and Afrotheria remains challenged [12]. In comparison, more distant branching events such as that of monotreme, marsupial and placental mammals can be significantly resolved with as little as 8000 aa sites of nuclear coding genes [14]. Molecular dating on phylogenomic data estimated that 30–40 Myr separated the splits among the three mammalian infra-classes [14]. This leaves enough time to accumulate phylogenetically relevant sequence differences and to reduce the effects of lineage sorting and prevents introgression.

While the monophyly of the clade Laurasiatheria is well established, phylogenomic analysis did not yet resolve the relationships within this clade. The internal branches of dog, cow, bat, and shrew received only 86% and 93% bootstrap support, respectively, in a recent phylogenomic analyses [3]. This non-significant support may be due to the "insufficient" amount of 200 knt of coding sequence that was available at the time of the analysis. However, the splits may also have occurred at a very narrow time window of a few millions years or less, and more data than expected may be required to resolve such tight divergences.

It is noteworthy that the few million years between mammal ordinal divergences are in the order of the average duration of mammalian species and their typical speciation times [15,16]. The fact that the splits between the extant orders occurred several tens of million years ago does not diminish the problem that these splits represent divergences among species or even lineages that were at the brink of becoming genetically separated species. Therefore, speciation adds an additional level of complication for resolving such narrow divergences. Not only is the time for such divergences relatively short to accumulate sufficient sequence differences that are needed to resolve these splits, but lineage sorting [17], species hybridization [18] and hybrid speciation [19] can make the resolution of significant parts of the mammalian tree all but impossible.

The continuously increasing amount of sequence data from protein coding genes now makes it possible to provide a phylogenomic analysis of placental mammals for 19 species on the basis of several million characters long alignments. The quantity of new genome data also allows using a new and more stringent assembly of the data, which aims at avoiding data from gene families and thus paralogous sequences in the alignment. We investigate if the resolution of the basal split among placental mammals [2] prevails with the increased taxon sampling and if other difficult to resolve divergences such as those within the Laurasiatheria and Euarchontoglires can be resolved with statistical confidence with an increased amount of phylogenomic data and new analytical approaches [12].

## Results

**Figure 1:**
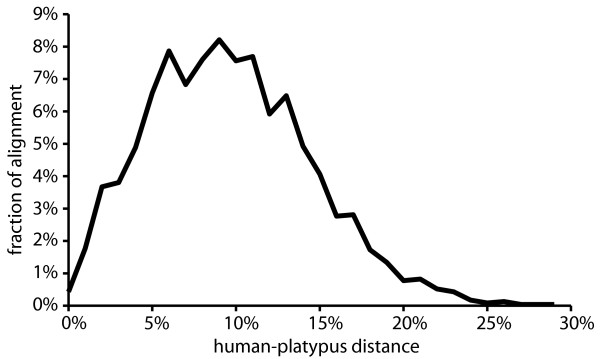
Distance distribution of the human-platypus aa sequence alignment.

The first step in the data collection, searching for human protein-coding genes that do not have obviously closely related members of a gene family resulted in a set of 14,302 human sequences, from the original 24,108 unique genes. These sequences were used for identifying related sequences from the remaining 21 species. After selecting against genes from multi-gene families in any of the species (step two) and using only alignments in which more than 16 species are represented, 8,813 multiple sequence alignments remained. From this data set sequence alignments that were shorter than 300 bp or observed aa distances (p) larger than 30% for any species pair were removed. This left a data set of 3,012 multiple sequence alignments. The total size of this alignment was 2,844,615 nucleotides for 22 species. The average length of the individual sequences is 944 ± 748 nt and the average p distance between human and platypus is 18.6 ± 4.32%, with a maximum of 30% (Figure 1), which is expected from the filtering constraints.

The rigorous filtering by recursive BLAST search removed sequences that are part of gene families notably reduced the amount of data. The filtering was rather effective, even though there is no formal proof for its efficiency yet. Already after the first filtering step (human sequence against human genome) sequences of the hemoglobin, myosin, olfactory receptor, HOX gene and other sequences that are part of known gene families were eliminated from the data set. It is conceivable that by this approach data from other, less known gene families were also excluded from the analysis. The recursive BLAST search with a cutoff value of 10^-12 ^identified genes in gene families that had more than 75% sequence similarity and eliminated these. It can be argued that more distant sequences will not be found when searching for such sequences in other species with the same cutoff value of 10^-12^.

When inspected by eye the base and amino acid frequencies appear to be very similar among the species. However, due to the large size of the dataset the chi-square test may become overly strict. Compositional homogeneity was rejected for many species both for nucleotide and amino acid data (see Table 1). The compositional heterogeneity remained even when the data were R/Y-coded.

**Table 1:** Nt composition (*f*) and chi-2 probabilities (P) for homogeneous character composition for nt, R/Y-coded and aa sequence data.

Species	*f*(A)	*f*(T)	*f*(G)	*f*(C)	*f*(R)	*f*(Y)	*P*(nt)	*P*(R/Y)	*P*(aa)
Human	27.6	24.0	24.7	23.8	52.2	47.8	0.00	1.33	49.1^a^
Chimpanzee	27.6	24.0	24.7	23.8	52.2	47.8	0.00	1.23	27.5^a^
Macaque	27.5	24.0	24.7	23.8	52.2	47.8	0.00	90.20^a^	90.3^a^
Galago	27.5	24.0	24.6	23.9	52.2	47.8	0.00	86.57^a^	4.10
Treeshrew	27.1	23.4	25.0	24.5	52.2	47.8	0.00	41.72^a^	91.6^a^
Rat	26.7	22.8	25.3	24.8	52.0	48.0	0.00	0.00	0.00
Mouse	26.8	23.2	25.3	24.7	52.1	47.9	0.00	0.37	0.02
Guinea pig	27.1	23.6	25.0	24.3	52.1	47.9	5.03^a^	33.09^a^	63.7^a^
Squirrel	27.5	24.1	24.6	23.9	52.1	48.0	0.00	1.19	30.4^a^
Rabbit	26.6	22.8	25.6	25.0	52.2	47.8	0.00	88.4^a^	0.01
Cat	26.5	22.9	25.6	25.1	52.0	48.0	0.00	0.00	0.00
Dog	27.2	23.6	24.9	24.2	52.1	47.9	0.00	28.22^a^	93.8^a^
Cow	26.7	23.0	25.4	24.9	52.1	47.9	0.00	1.42	0.04
Microbat	26.5	22.8	25.6	25.2	52.1	47.9	0.00	0.07	0.00
Shrew	26.7	23.0	25.4	25.0	52.0	48.0	0.00	0.05	40.7^a^
Hedgehog	27.2	23.6	24.9	24.4	52.1	47.9	0.00	0.10	48.9^a^
Elephant	27.4	23.8	24.8	23.0	52.2	47.8	0.00	68.44^a^	7.28^a^
Tenrec	26.3	22.5	25.7	25.5	52.0	48.0	0.00	0.00	0.00
Armadillo	27.5	23.8	24.7	24.0	52.2	47.8	0.00	71.50^a^	77.3^a^
Opossum	28.4	24.9	23.8	22.9	52.2	47.8	0.00	35.09^a^	0.00
Platypus	26.5	22.3	25.7	25.5	52.2	47.8	0.00	17.3^a^	0.00
Chicken	28.8	24.5	24.3	22.5	53.0	47.0	0.00	0.00	0.00

^a ^Species for which compositional homogeneity could not be rejected with 5% significance, and are thus assumed to be homogeneous.

The evolutionary model and the number of rate heterogeneity classes were estimated from previous phylogenomic analyses [2]. Exact tests such as implemented in MODELTEST [20] could not be performed due to computational constraints and the inability of most programs to analyze a data set of this magnitude. A manual analysis would have been prohibitively time consuming to perform and we suspect would have resulted in the same model selection.

**Figure 2:**
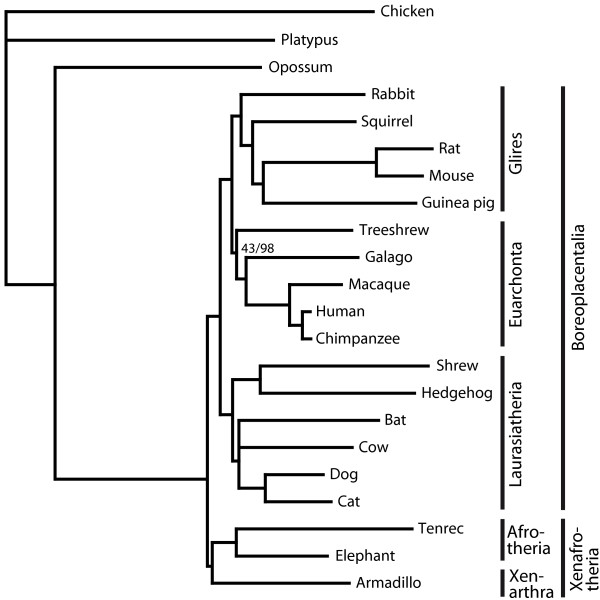
ML tree on NT12 analysis and ML bootstrap support values (nt/aa) for branches that do not receive maximum support.

The phylogenetic analyses of the data produced an unambiguous picture of most placental mammals relationships. The ML tree in Figure 2 was constructed from first and second codon positions (NT12) and analyzed under a GTR 8Γ+I model of sequence evolution for the 2,844,615 nt long sequences in TF. Although in this and most other analyses the bat groups with the cow, the branch is shown as unresolved in Figure 2, because the placement of the bat on the mammalian tree is indefinite in some analyses. Bayesian analysis of the same data set, on first plus second codon positions or on aa sequence data reconstructed the same topology. Also R/Y coded sequences that were analyzed using a two-state [21] ML 4Γ+I model reconstructed the topology shown in Figure 2. ML analysis with a non-stationary model in PAML [22] confirmed the topology in Figure 2. Finally, the same tree was also reconstructed after removing sites with more than three different amino acids across all species.

**Figure 3:**
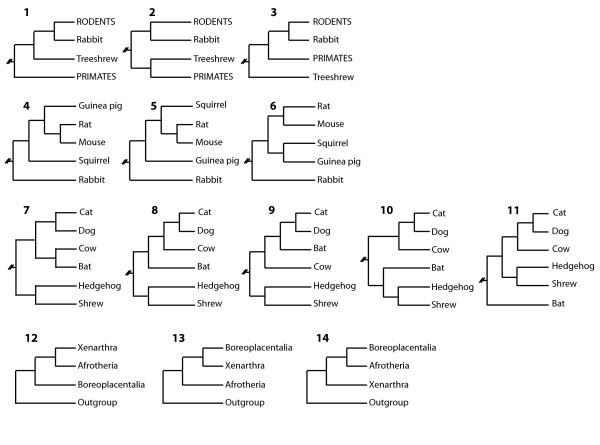
Partial trees illustrating the alternative topologies that were statistically evaluated to determine the phylogenetic position of the tree shrew (1–3), the relationships within Rodentia (4–6), the position of the bat (7–11), and the Xenarthra, Afrotheria and Boreoplacentalia relationships (12–14).

Placing the treeshrew on the mammalian tree by sequence analysis was somewhat problematic. ML reconstructed an alternative position of the treeshrew, outside the primates plus Glires clade, when the nt data were not partitioned by codon position. An extended ML analysis on aa and first and second codon position sequences (NT12) could, however, differentiate between alternative placements of the treeshrew on the tree (Table 2a, Figure 3, tree 1–3) for the different datasets. A sister group position to the primates is the best ML option for most analyses. The treeshrew as sister group to primates plus Glires can be rejected by other datasets with statistical confidence. Also the grouping of the treeshrew with the Glires can be rejected by ML analysis of aa and NT12. Neither a codon analysis of the concatenated dataset or a separate analysis of 402 individual genes, a total of 501,012 nt of sequence data, for which full taxon sampling was available, clarified the position of the treeshrew, because none of the topologies in Table 2a received significant support.

**Table 2:** Support for alternative positions (see fig. 3) of controversial relationships of treeshrew (a), guinea pig (b), bat (c), and Boreoplacentalia, Afrotheria and Xenarthra (d). SH probabilities are shown for analyses of concatenated sequences and bootstrap probabilities are shown for the separate analyses of individual sequences (marked: sep).

a)							
	AA	NT123	NT12	NT123 cdp	codon	NT123 sep	AA sep

Tree 1	0.0286	0.4355	0.013	0.1077	0.613	0.1853	0.1320
Tree 2	**1.0**	0.6312	**1.0**	**1.0**	**1.0**	**0.8146**	0.3325
Tree 3	0	**1.0**	0	0	0.048	0.0001	**0.5355**

b)							

	AA	NT123	NT12	NT123 cdp	codon	NT123 sep	AA sep

Tree 4	**1.0**	**1.0**	**1.0**	**1.0**	**1.0**	**1.0**	**1.0**
Tree 5	0	0.0002	0	0	0	0	0
Tree 6	0	0	0	0	0	0	0

c)							

	AA	NT123	NT12	NT123 cdp	codon	NT123 sep	AA sep

Tree 7	**1.0**	**1.0**	**1.0**	**1.0**	**1.0**	**0.9512**	0.2728
Tree 8	0.003	0	0	0	0	0	0
Tree 9	0	0	0	0	0	0	0
Tree 10	0.625	0	0	0	0.182	0.0488	**0.7273**
Tree 11	0	0	0	0	0	0	0

d)							

	AA	NT123	NT12	NT123 cdp	codon	NT123 sep	AA sep

Tree 12	0.5854	**1.0**	**1.0**	0.0486	**1.0**	**0.7549**	0.0346
Tree 13	**1.0**	0.0040	0	0.0442	0.094	0.0170	0.2342
Tree 14	0.6176	0.0458	0	**1.0**	0.079	0.2281	**0.7312**

Alternative phylogenetic positions of the guinea pig among the rodents can clearly be rejected by an SH test on all analyzed data types (Table 2b, Figure 3, tree 4–6).

The highly unconventional phylogenetic position of the bat as sister group to the cow and thus probably to all Artiodactyla and Cetacea is recovered by most analyses and datasets (Table 2c, Figure 3, tree 7–11). The only alternative position is as sister group to the lipotyphla (hedgehog plus shrew) which cannot be rejected by the SH test on aa or codon sequence data. It is the favored topology for separate aa analysis of individual genes. Albeit, the support is very limited. Varied results were obtained from ML analyses on data that were partitioned according to the average aa distance. Without following logic the bat grouped either with the cow or the shrew plus hedgehog. Analyses of partitions with over 10% aa distance grouped the bat indistinguishably by ML on any of the neighboring branches. Although most analyses favor a grouping of bat plus cow, the branch leading to the bat, cow and carnivores is shown as unresolved in Figure 2.

ML analyses of all nt sequences (NT123), NT12, codon model analysis and separate analysis of nt sequences favor a split between Xenafrotheria and the remaining placentals (Table 2d, tree 12). However, analysis of aa sequences ever so slightly favor a split between Afrotheria and the remaining placentals (Table 2d, tree 13), but cannot reject alternative hypothesis. Partitioned ML analysis of codon positions and separate analysis of aa sequences support a split between Xenarthra and the remaining placentals (Table 2d, tree 14).

ML bootstrap support values for the branches that do not receive maximum support are shown in Figure 2. Thus, bootstrap analysis unambiguously supports the relationships among Xenafrotheria, Glires and primates. As expected, nt sequences find only limited support for the treeshrew as sister group to the primates, but even the analysis of aa sequences does not provide maximal bootstrap support for this relationship. While nt sequences provide the strongest bootstrap support (91%) for placing the cow and bat on a common branch, ML bootstrap analysis of aa sequences data support the bat plus cow grouping only by 31% and instead favors to group the bat with the shrew plus hedgehog on a common branch. This relationship receives 64% bootstrap support.

**Figure 4:**
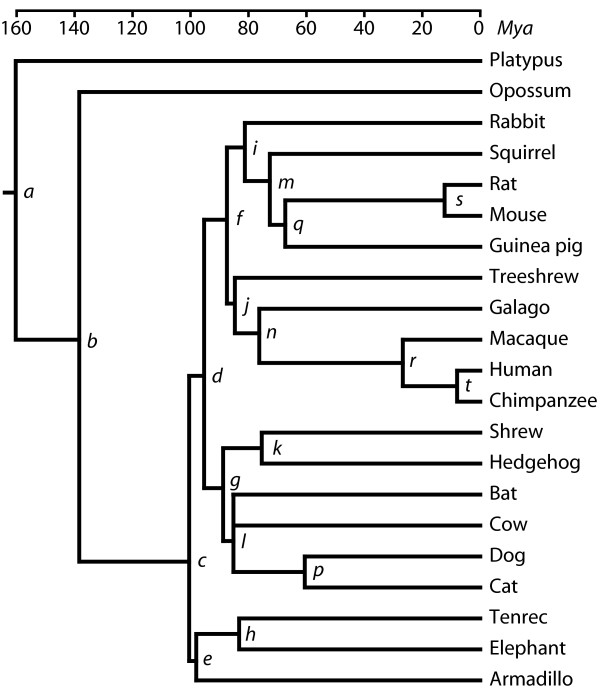
**Chronogram showing the estimated divergence times.** The figure is based on divergence times estimated from nt sequences using TF. Divergences are labeled with letters (a-t) and the exact dates and the values estimated by other methods and sequence data are shown in Table 3.

Divergence times were calculated for the tree topology as shown in Figure 1. Estimated ages for all divergences are depicted in a chronogram in Figure 4. The exact values for different methods and their standard deviations for the r8s dates based on aa and nt sequences are shown in Table 3. Virtually the same divergence times among Boreoplacentalia were calculated for the two alternatively rooted trees (Table 4). For some dates the r8s program tend to provide 4–5 Myr younger time estimates than TF. However, the dates provided by TF generally conform within a few 100,000 years to those previously published on phylogenomic analyses [2], and most importantly, only marginal differences are observed for the relative dates. The standard deviations appear to be unrealistically small, but reflect the amount of data that was used for the analysis. Most divergence time estimates are only negligibly different from analysis of much smaller nuclear or mitogenomic alignments [23]. A notable exception is the age of the lagomorph and rodent divergence, which has previously been estimated 5–20 Myr younger by phylogenomic analysis [2]. It should be observed that many of the difficult to resolve divergences are splits that are separated by only a few Myr. These involve e.g. the treeshrew, bat, primate, Glires, and the Boreoplacentalia and Xenafrotheria divergence.

**Table 3:** Divergence time estimates for branches *a-t *in figure 4, were based on aa and nt sequences using TF and r8s.

Divergence	TF aa (Mya)	TF nt (Mya)	r8s aa (Mya)	r8s nt (Mya)
*a*	160.3	162.3	160.22 ± 0.34	160.34 ± 0.27
*b*	138.4	138.4	138.4 ± 0.00^*b*^	138.4 ± 0.00^*b*^
*c*	100.5	99.93	95.0 ± 0.00^*b*^	95.0 ± 0.00^*b*^
*d*	95.3	95.31	89.74 ± 0.16	89.57 ± 0.07
*e*	98.03	97.85	93.20 ± 0.30	93.20 ± 0.07
*f*	87.44	88.4	81.67 ± 0.18	80.91 ± 0.14
*g*	88.75	88.86	83.61 ± 0.18	83.21 ± 0.09
*h*	83.21	79.74	77.69 ± 0.24	77.56 ± 0.17
*i*	81.24	83.47	75.88 ± 0.19	74.86 ± 0.19
*j*	84.69	86.53	79.96 ± 0.22	79.00 ± 0.18
*k*	75.41	76.25	71.55 ± 0.21	71.29 ± 0.11
*l*	85.18	85.87	80.94 ± 0.30	80.44 ± 0.12
*m*	72.62	74.04	64.45 ± 0.17	63.46 ± 0.25
*n*	76.27	78.53	72.69 ± 0.21	71.47 ± 0.18
*o*^*a*^	80.11	81.1	76.36 ± 0.29	76.00 ± 0.15
*p*	60.56	59.98	57.17 ± 0.28	56.50 ± 0.11
*q*	67.3	69.62	60.31 ± 0.18	59.47 ± 0.26
*r*	26.73	28.88	26.76 ± 0.20	26.21 ± 0.12
*s*	12.3	12.3	12.3 ± 0.00^*b*^	12.3 ± 0.00^*b*^
*t*	9	9	7.85 ± 0.15	7.67 ± 0.10

^*a *^refers to the cow-bat branching which is shown unresolved in figure 4.

^*b*^Estimated date is on the edge of the calibration interval and thus no standard deviation could be calculated.

**Table 4:** Divergence time estimates from nt sequences using TF, for trees with alternative placements of Xenarthra and Afrotheria (trees 12–14 in figure 3).

Divergence	Tree 12	Tree 13	Tree 14
*a*	162.3	161.6	161.8
*b*	138.4	138.4	138.4
*c*	99.93	ND	ND
*d*	95.31	95.31	95.30
*e*	97.85	ND	ND
*f*	88.4	87.82	87.74
*g*	88.86	88.44	88.64
*h*	79.74	88.15	83.1
*i*	83.47	82.76	82.65
*j*	86.53	85.81	85.98
*k*	76.25	75.64	75.91
*l*	85.87	85.41	85.63
*m*	74.04	73.38	73.29
*n*	78.53	77.87	77.69
*o*^*a*^	81.1	80.66	80.82
*p*	59.98	59.51	59.68
*q*	69.62	68.97	68.89
*r*	28.88	27.4	27.38
*s*	12.3	12.3	12.3
*t*	9	9	9

The branches *c *and *e *do not exist and were not determined (ND).

^*a *^refers to the cow-bat branching

## Discussion

The study of placental mammal relationships has drawn the attention of generations of scientists. However, the resolution of the mammalian tree has been problematic and many relationships remained unresolved or only poorly supported. Initially phylogenetic studies were made on morphological data from recent and fossil species [8,24-26], immunological data [27], sequence data of single genes [28], sequence data from whole mitochondrial genomes [29], concatenation of several nuclear encoded genes [6], hundreds of nuclear genes from expressed sequence tag data [14,30], and now phylogenomic analyses, which cover 10% of the coding region of the genome. The use of rare genomic characters such as retroposed elements [7] or indels [11] as cladistic markers has become an important addition to the analysis of sequence data.

The steadily increasing amount of genomic sequence data from placental mammals does not only allow an ever-deeper insight into their evolution, but it also allows development of rigorous data handling procedures. A central aim in all phylogenomic analyses is selecting orthologous sequences for data analysis. The lack of complete chromosome maps for most genomes make it currently impractical to utilize synteny information and orthology can only be established when the species phylogeny is known [31]. However, the mammalian phylogeny is often unclear and the very issue of such studies. Therefore, in most phylogenetic analyses orthology is determined by a sequence similarity criterion [6,32] or choosing from a pre-determined database of orthologous sequences, such as COG [33] and the orthologs matrix project, OMA [34]. These databases usually use similarity criteria too and are sometimes combined with manual curation for increasing the confidence in the orthology of the sequences.

In the mammalian genome, the ratio of gene to gene family is about two and the size of about half of the gene families have changed during mammalian evolution [35]. The globin genes are a textbook example of such gene families [36]. The human beta hemoglobin family has six members that have been duplicated at different times during evolution of mammals and they fulfill different functions. Other mammals, such as goat and sheep, which have different history of gene duplications, have as many as 13 members of the beta hemoglobin family [37]. Thus, there is a considerable risk that the orthology of sequences from gene families cannot be securely established, unless by laborious case-to-case studies for each gene and each species involved. Such an approach is prohibitive for large-scale phylogenetic analyses.

The rigorous approach of the data assembly, recursive BLAST, in our analysis excludes the vast majority of sequences from multi-gene families, which are the source of paralogous sequences, from the analysis. Already the first filtering step successfully removed sequences from typical gene families. While this is no formal proof that all genes from gene families were removed, it is conceivable that other, less thoroughly studied, gene families were also eliminated from the analysis during this process. Thus, the risk for including paralogous sequences in the current analysis was greatly reduced by the recursive BLAST approach.

The data collection and filtering produced a 2.8 Mbp long dataset composed of 3012 alignments of inferred transcripts from genes for 21 mammalian and one avian species with a moderate evolutionary rate. This alignment is well suited for studying deep phylogenetic divergences in the placental tree. The compositional bias of the data remained to be a major obstacle in the analysis, which could not be overcome for most sequences by recoding the sequences to R/Y. However, congruency of the reconstructed trees with analyses using a non-stationary model, indicate that compositional bias does not influence the tree topology to a greater extend. Further and less well-defined systematic errors are a general threat to phylogenetic analysis [38] and are the source for strong support for erroneous relationships. Removing sites with more than three different aa, i.e relatively variable sites, or analyzing different data partitions according to evolutionary rate are a recommend cure to the problem. However, these analyses did not change the topology. The taxon sampling currently available for genome analysis does not yet allow for investigating if one or several rouge taxa influence the ML reconstruction.

The reconstructed phylogeny is largely congruent with that of previous studies. Most analyses favor a basal divergence of Xenafrotheria and Boreoplacentalia. This split is supported by phylogenomic sequence analyses, two retroposon insertions and indels [2,4,11]. The revised naming of the Boreoeutheria clade is preferable to the previously used term, because of the improved consistency and logic. Recent naming of new clades were largely based on molecular phylogenetics, which by definition exclude the stem group members of a clade. Thus, the term "eutheria" in the formerly used Boreoeutheria encompasses also stem group eutherians only known from the fossil record. In comparison "placentalia" describes the respective clade more accurately, referring only to crown group members, which are accessible for molecular studies [13].

The elephant and tenrec group, as expected, in the Afrotheria clade [9]. The Boreoplacentalia are split into Laurasiatheria and Eurchontoglires, the latter with a well-supported Glires clade. Within the rodents the guinea pig fell between Myomorpha (rat and mouse) and Sciuromorpha (squirrel), which is consistent with some previous findings of partially constraint tree analyses [6], but inconsistent with some mitogenomic studies, which find the squirrel (Sciuridae) closer to the muroid mouse and rat to the exclusion of the guinea pig [39].

The phylogenetic position of the treeshrew (Scandentia) within the Euarchontoglires was problematic even when analyzing nearly 3 Mnt of protein coding data. The uncertainty of placing the treeshrews among placental mammals is evident from the numerous positions it had been placed on the tree in previous studies. The treeshrews have been placed as the first divergence among Euarchontaglires [40,41], as sister group to Lagomorpha or rodents [42], sister group to Dermoptera [6], sister group to primates [5] or part of the traditional Archonta clade outside a ((Primates, Dermoptera), Chiroptera) clade [43]. Two recent studies on rare genomic changes, found support for a grouping the treeshrew with primates, from four indels [40] and five retroposed elements [41] respectively. This conforms with our sequence analysis based findings.

The placement of the bat as sister group to the cow in most ML analyses is highly unexpected and has not been proposed in any other analyses. Currently the result can only be regarded as peculiar. The only alternative place of the bat that cannot be rejected by ML analysis of aa sequences is on a common branch to the Lipotyphla (hedgehog+shrew). This grouping receives inconclusive 64% bootstrap support. Mitogenomic and nuclear gene data generally find the bat (Chiroptera) as the sister group to Cetferungulata [3,6,44], which is the currently preferred hypothesis. The notable exception to the general consensus came from a recent retroposon analysis that found support for a sister group relationship of Chiroptera to Perissodactyla plus Carnivora [45]. If this particular relationship was supported by our sequence data, the bat would have been placed on the carnivore branch, because there is no species from Perissodactyla present in our data set. However, ML analyses can exclude a relationship of the bat and carnivores and most alternative positions with high statistical confidence.

Systematic errors can never be formally excluded as a cause for reconstructing inaccurate relationships. Therefore we have investigated the effect of two major sources of systematic error, namely highly variable sites and highly variable sequences [38]. The exclusion or separate analyses of these had no clear consequence on the reconstructed topology. Until genome data from further species become available for analysis, we regard the phylogenetic position of the bat as unsettled by genomic sequence data.

The divergences among the placental mammal orders in this study occurred between ≈100 and ≈80 Mya, thus well inside the Cretaceous (140–65 Mya) and agrees with previous molecular based estimates on mitogenomic and phylogenomic data [2,11,23] but is in steep contrast with some recent interpretations of the fossil record [46]. Many ordinal and higher level divergences occur within a few Myr from each other. TF estimates the Xenafrotheria and Boreoplacentalia divergence ≈100 Mya and the first split within these clades is estimated to ≈98 Mya and ≈95 Mya. The r8s estimates are somewhat younger, but present the same picture. Similarly, the Euarchontoglires clade diverges at ≈87 Mya into Glires and Euarchonta, and at ≈85 Mya into treeshrew (Scandentia) and the primate lineage. These splits are remarkably close to each other and would prove problematic to resolve even from recent species. One major problem of resolving short divergence time intervals is that an insufficient amount of phylogenetic information accumulates in these short intervals. This obstacle can usually be overcome simply by increasing the amount of data, which is the current strategy in phylogenomic analyses.

The other, more severe, problem that cannot as easily be solved is connected to genomic processes around speciation. Before closely related species become completely genetically isolated they can hybridize with each other. This poses a problem for phylogenetic analysis, because the species history can be obscured by introgression, the incorporation of genes from one species into the gene pool of another. It has been estimated that such hybridization can occur up to 7–17 Myr after separation in birds [47] and thus probably also in mammals. Cattle-zebu (*Bos taurus – Bos indicus*) hybrids are common and fully fertile despite that the respective lineages have been separated for 1–2 Myr [48]. There are surprisingly many examples where viable, fertile and even well adapted offspring form by species hybridization in mammals [18,49,50]. It has been estimated that 10% of all animals (6% of European mammals), actually hybridize with at least one other species [47]. Many more may have gone through a phase of hybridization that remains undetected, because the species that now are observed to hybridize are usually the evolutionarily youngest.

Another process that can obscure phylogenetic analysis is lineage sorting. Lineage sorting is the result of an ancestral polymorphism that survives a speciation event, succeeded by fixation of different alleles in the descendant lineages. This gives rise to a gene history that is incongruent with the species' history [17]. The extent to which lineage sorting jumbles the genome of closely related species has recently been investigated for the anthropoid genomes, i.e. the genomes of human, chimpanzee, gorilla, and orangutan. For about one quarter of the human genome gorilla and not chimpanzee is the closest genetic relative [51]. In 0.04% of the studied genes the human sequence share closest ancestry even with the orangutan. Lineage sorting is currently the favored hypothesis to explain the mosaic of the primate genomes [51,52]. However, other natural processes such as hybridization have been used to explain the fact that some parts of the genome are more similar between human and gorilla than between human and chimpanzee [53,54].

Finally, a process with a similar effect on the genome as species hybridization is hybrid speciation [19], which may be a new and radical explanation for the concoction of the mammalian genome. In this process two previously isolated sister-species hybridize and give offspring to a new and possibly better-adapted species [19]. While such a process is common in plants, there is growing evidence that this also occurs in animals, including vertebrates [50,55].

Which of these processes, lineage sorting or hybridization, has the dominating effect on the mammalian genome cannot be determined yet and needs to be studied in more detail. In any case, these processes seem to have a sizeable impact on the resolution of short internal branches of the mammalian tree and phylogenetic reconstruction in general. The studies of the jumbled anthropoid genomes [51,53,54] are the first to put some exact values on the amount of gene trees that are incongruent with the species tree.

It must be recognized that there is a non-negligible chance that a fair amount of single loci have a history that is not compatible with the species history. Therefore introgression or linage sorting are problematic especially to super-tree analyses [56] and analyses from rare genomic events [57]. In these approaches of phylogenetic reconstruction many branches are only supported by single or at most a few genes or loci. Incompatibilities in the support from the analysis of retroposon insertion for basal and ordinal mammalian divergences [7,11,45] probably document introgression or linage sorting events that confound the genome history. Probably the only solution is the collection of large amounts of information as in phylogenomic analyses in the hope that the massive amount of data evens out events that lead to a genome history that is inconsistent with the species history. Large phylogenomic analyses produce a tree that, at least on average, reflects the species history.

Completely or nearly completely sequenced genome data are becoming available for phylogenomic analysis of mammalian relationships at an increasing rate. This allows ever more detailed reconstruction of their history and their major branches are becoming with time more consistently recovered. However, some mammalian relationships will require more detailed studies of their history, taking into account that the genome is not a fixed entity but malleable by speciation events.

## Conclusion

Phylogenomic analysis of 3,012 genes (2,844,615 nucleotides) from 19 placental mammal species could significantly resolve most relationships and date their basal divergences in the Cretaceous at 100 – 90 Mya. However, the mammalian divergences that occurred in very narrow time windows of 2–4 Myr remain surprisingly difficult to resolve, even by the huge amount of genomic data. Divergences of lineages that are now considered to be orders, started as speciation events even if they occurred millions of years ago. Recent species that have been separated for about the same amount of time can still hybridize and have fully fertile offspring, leading to introgression, or may be affected by lineage sorting that results in a gene history that is different from the species history. Thus, some deep mammalian divergences that are separated by only a few Myr appear to be affected by speciation processes that obscure the phylogenetic reconstruction.

## Methods

Predicted cDNA sequences from chicken and all mammalian genomes with assemblies and gene builds in release 46 of ENSEMBL were downloaded from ftp://ftp.ensembl.org/pub/current_fasta/. In total 22 species were included, *Homo sapiens *(human), *Pan troglodytes *(chimpanzee), *Macaca mulatta *(macaque), *Otolemur garnettii *(galago), *Tupaia belangeri *(treeshrew), *Rattus norvegicus *(Norway rat), *Mus musculus *(house mouse), *Cavia porcellus *(guinea pig), *Spermophilus tridecemlineatus *(ground squirrel), *Oryctolagus cuniculus *(rabbit), *Felis catus *(cat), *Canis familiaris *(dog), *Bos taurus *(cow), *Myotis lucifugus *(little brown bat), *Sorex araneus *(shrew), *Erinaceus europaeus *(western european hedgehog), *Loxodonta africana *(African elephant), *Echinops telfairi *(tenrec), *Dasypus novemcinctus *(armadillo), *Monodelphis domestica *(opossum), *Ornithorhynchus anatinus *(platypus) and *Gallus gallus *(chicken). These species represent 12 of the 21 extant eutherian orders. One species from each of Metatheria (marsupials) and Prototheria (monotremes), and finally Aves (bird) served as outgroups to the placental mammals.

In order to avoid including paralogous sequences in the alignment we developed a new search strategy that aims at excluding sequences that stem from recent gene duplications. This approach can identify and eliminate duplicated sequences that exist in several rather similar copies within each genome. This decreases the risk of including paralogous sequences in the analysis.

The first step of the data collection process is a "recursive BLAST search". It was conducted by searching each human sequence against all human sequences using BLAST [58] with a cutoff E-value of 10^-12^. Sequences that had identified transcripts originating from more than one gene were excluded from further investigation. In cases where several transcripts of different lengths from the *same *gene were found, supposedly representing different splicing variants, only the longest sequence was retained.

In the second step, human sequences that passed the criteria of the first step were used for searching for similar genes in other species. The human sequences were used in a BLAST search against each of the species' databases with a cutoff E-value of 10^-12^. The sequence with the highest E-value score was kept for further examination. In the third step, the sequence from step two was used for a recursive BLAST search with a cutoff E-value of 10^-12 ^against the same species. Similar to step one, only sequences with a single hit were kept for further analysis.

Finally, genes for which sequences could be found for fewer than 16 of the 22 species were removed. Sequences shorter than 300 bp were also removed. The remaining sequences were translated to amino acids and multiple sequence alignments were created using MUSCLE [59]. Sequences with more than 30% observed (p) distance at the aa level for any species pair were removed. The aa alignments were then used as a template for creating nucleotide sequence alignments using the original, untranslated sequences.

Prior to the phylogenetic reconstruction the data set was analyzed for identifying compositional biases and other anomalies that could possibly influence the tree reconstruction. A chi-square test on compositional homogeneity was performed on the base frequencies, R/Y recoded nucleotide sequences, (A and G sites were coded R (purine) and T and C were coded to Y (pyrimidine)) and aa sequences. In addition the sequence data were analyzed by maximum likelihood (ML) using the PAML v.3.15 program baseml [22] with a non-stationary model (nhomo = 3), that allows for different base composition on different branches [60]. The R/Y recoding and ML analysis with a non-stationary model was done for detecting reconstruction artifacts that may be caused by compositional biases, but did not replace the standard ML analysis.

The alignment was also inspected for any grossly deviating distances between human and platypus that might have escaped detection during the data collection process. The p distances between human and platypus were calculated in a sliding window of 800 amino acids, that was moved 400 steps for each calculation until the end of the concatenated alignment was reached. These two species were arbitrarily chosen to represent one of the most distant mammal species pairs in this analysis and for one (human) of which the most complete genomic data are available.

Due to the size of the data set most programs were apparently not able to analyze the fully concatenated data properly. ML analyses could be performed on the full alignment with Treefinder version June 2007 [61] only. Other programs such as TREE-PUZZLE [62], PHYML [63] or PAML either crashed or required a seemingly infinite amount time to finish a single analysis. For the ML analysis with PAML the data were randomly partitioned into manageable data packages and the results combined with the "totalml" program from PHYML. The nt tree was reconstructed by TF with the GTR model [64] with rate heterogeneity assuming eight classes of gamma distributed rate categories [65] and one class of invariable sites (8Γ + I). The analyses of aa sequences were performed using the WAG2000 model [66] applying rate heterogeneity (8Γ + I). Uncertain or controversial relationships were further analyzed by an extended ML analysis where different possible topologies were statistically evaluated in TF using Shimodaira-Hasegawa probabilities, pSH [67]. For these comparative analyses an additional analysis with a codon substitution model was performed in PAML with rate heterogeneity (8Γ). ML Bootstrap support values were calculated from 100 replicates of aa and nt sequences. The analysis was done on the Bioportal cluster on University of Oslo running TF for 28 days.

The data were randomly divided into several partitions of approximately 300,000 nt (100 kaa) for Bayesian inference using MrBayes 3.1.2 [68], running for 1,000,000 Markov chain Monte Carlo generations with one cold and three heated chains, discarding the first 100,000 generations as burn-in and then sampling each 100th tree. R/Y coded sequences that were analyzed using a two-state ML model [21] with (4Γ+I). Partitioned analysis was performed in TF and MrBayes with data partition according to codon positions. The data were also partitioned into alignments with an overall aa distance of 2%, 4%, 6%, 8%, 10% and >12% and analyzed separately by ML. Finally the data were also analyzed by ML after removing sites with more than three different aa sites.

In addition to the analysis of the concatenated data we analyzed alignments from individual genes with full taxon sampling (402 alignments, 501,012 bp) separately with PAML, both for nt and aa sequence data. The likelihood values were then combined with totalml [63] and the bootstrap probabilities were recorded.

Divergence times were estimated from both aa and nt sequence data using the nonparametric rate smoothing method on a logarithmic scale (NPRS-LOG) implemented in TF and using the r8s program [69] applying the NPRS method and the POWELL algorithm. The eight fossil-based age constraints that were used to calibrate the tree were taken from Benton & Donoghue [70] and are listed in Table 5. Mean values and their standard deviations were calculated in r8s from the branch lengths of 100 bootstrapped ML analyses of aa and nt sequences.

**Table 5:** Calibration points used for dating placental mammal divergences.

Split		Minimum age (Mya)	Maximum age (Mya)
Eutheria	Metatheria	125.2	138.4
Boreoplacentalia	Xenafrotheria	95.3	113
Euarchontoglires	Laurasiatheria	95.3	113
Lagomorpha	Rodentia	61.5	100.5
Caniformia	Feliformia	43	63
Apes	Old World monkeys	23	34
Rat	Mouse	11.0	12.3
Human	Chimpanzee	6.5	10

## Authors' contributions

BMH performed data collection and preparation, and conducted the phylogenetic analyses. AJ had the initial idea. AJ and BMH wrote the manuscript.

## References

[B1] Cannarozzi G Schneider A Gonnet G A phylogenomic study of human, dog, and mouse PLoS Comput Biol 2007 3 1e2 10.1371/journal.pcbi.0030002 PMC1761043 17206860

[B2] Hallström BM Kullberg M Nilsson MA Janke A Phylogenomic data analyses provide evidence that Xenarthra and Afrotheria are sister groups Mol Biol Evol 2007 24 2059 2068 10.1093/molbev/msm136 17630282

[B3] Nikolaev S Montoya-Burgos JI Margulies EH Program NCS Rougemont J Nyffeler B Antonarakis S Early History of Mammals Is Elucidated with the ENCODE Multiple Species Sequencing Data PloS Genet 2007 3 1 10.1371/journal.pgen.0030002 PMC1761045 17206863

[B4] Wildman DE Uddin M Opazo JC Liu G Lefort V Guindon S Gascuel O Grossman LI Romero R Goodman M Genomics, biogeography, and the diversification of placental mammals Proc Natl Acad Sci USA 2007 104 14395 14400 10.1073/pnas.0704342104 17728403 PMC1958817

[B5] Novacek MJ Mammalian phylogeny: shaking the tree Nature 1992 356 121 125 10.1038/356121a0 1545862

[B6] Murphy WJ Eizirik E O'Brien SJ Madsen O Scally M Douady CJ Teeling E Ryder OA Stanhope MJ de Jong WW Springer MS Resolution of the early placental mammal radiation using Bayesian phylogenetics Science 2001 294 2348 2351 10.1126/science.1067179 11743200

[B7] Kriegs JO Churakov G Kiefmann M Jordan U Brosius J Schmitz J Retroposed elements as archives for the evolutionary history of placental mammals PLoS Biol 2006 4 4e91 10.1371/journal.pbio.0040091 PMC1395351 16515367

[B8] McKenna MC Bell SK Classification of mammals – above the species level 1997 New York: Columbia University Press

[B9] Stanhope MJ Waddell VG Madsen O de Jong W Hedges SB Cleven GC Kao D Springer MS Molecular evidence for multiple origins of Insectivora and for a new order of endemic African insectivore mammals Proc Natl Acad Sci USA 1998 95 9967 9972 10.1073/pnas.95.17.9967 9707584 PMC21445

[B10] Asher RJ Rose KD, Archibald JD Insectivoran-grade placental mammals: character evolution and fossil history The Rise of Placental Mammals 2005 Baltimore and London: The Johns Hopkins University Press 50 70

[B11] Murphy WJ Pringle TH Crider TA Springer MS Miller W Using genomic data to unravel the root of the placental mammal phylogeny Genome Res 2007 174 413 421 10.1101/gr.5918807 17322288 PMC1832088

[B12] Nishihara H Okada N Hasgawa M Rooting the eutherian tree: the power and pitfalls of phylogenomics Genome Biol 2007 8 R199 10.1186/gb-2007-8-9-r199 17883877 PMC2375037

[B13] Arnason U Adegoke J Gullberg A Harley E Janke A Kullberg M Mitogenomic Relationships of Placental Mammals and Molecular Estimates of their Divergences Gene in press 10.1016/j.gene.2008.05.024 18590805

[B14] Kullberg M Hallström B Arnason U Janke A the platypus reject the Marsupionta and acknowledge the Theria hypothesis Zoologica Scripta 2008 37 115 127 10.1111/j.1463-6409.2007.00319.x

[B15] Curnoe D Thorne A Coate JA Timing and tempo of primate speciation J Evol Biol 2006 19 59 65 10.1111/j.1420-9101.2005.00989.x 16405577

[B16] van Dam JA Abdul Aziz H Alvarez Sierra MA Hilgen FJ Hoek Ostende LW van den Lourens LJ Mein P Meulen AJ van der Pelaez-Campomanes P Long-period astronomical forcing of mammal turnover Nature 2006 443 687 691 10.1038/nature05163 17036002

[B17] Nei M Molecular evolutionary genetics 1987 New York: Columbia University Press

[B18] Gray AP Mammalian hybrids: a check-list with bibliography 1972 2 Commonwealth Agricultural Bureaux

[B19] Mallet J Hybrid speciation Nature 2007 446 279 283 10.1038/nature05706 17361174

[B20] Posada D Crandall KA Modeltest: testing the model of DNA substitution Bioinformatics 1998 14 817 818 10.1093/bioinformatics/14.9.817 9918953

[B21] Felsenstein J Evolutionary trees from DNA sequences: a maximum likelihood approach J Mol Evol 1981 17 368 376 10.1007/BF01734359 7288891

[B22] Yang Z PAML: A program package for phylogenetic analysis by maximum likelihood Comput Appl Biosci 1997 13 555 556 9367129 10.1093/bioinformatics/13.5.555

[B23] Arnason U Janke A Mitogenomic analyses of eutherian relationships Cytogenet Genome Res 2002 96 20 32 10.1159/000063023 12438776

[B24] Owen R On the anatomy of vertebrates 1866 I and II Longmans, Green, London

[B25] Gregory WK The orders of mammals Bull Am Mus Nat Hist 1910 27 1 524

[B26] Simpson GG The principles of classification and a classification of mammals Bull Am Mus Nat Hist 1945 85 1 272

[B27] Nutall GHF Blood immunity and blood relationship 1904 Cambridge: Cambridge University Press

[B28] Goodman M Czelusniak J Beeber JE Phylogeny of primates and other eutherian orders: a cladistic analysis using amino acid and nucleotide sequence data Cladistics 1985 1 171 185 10.1111/j.1096-0031.1985.tb00420.x 34965670

[B29] Janke A Feldmaier-Fuchs G Thomas WK von Haeseler A Pääbo S The marsupial mitochondrial genome and the evolution of placental mammals Genetics 1994 137 243 256 8056314 10.1093/genetics/137.1.243 PMC1205941

[B30] Kullberg M Hallström B Arnason U Janke A Expressed sequence tags as a tool for phylogenetic analysis of placental mammal evolution PLoS ONE 2007 2 e775 10.1371/journal.pone.0000775 17712423 PMC1942079

[B31] Zmasek CM Eddy SR RIO: Analyzing proteomes by automated phylogenomics using resampled inference of orthologs BMC Bioinformatics 2002 3 14 10.1186/1471-2105-3-14 12028595 PMC116988

[B32] Remm M Storm CE Sonnhammer EL Automatic clustering of orthologs and inparalogs from pairwise species comparisons J Mol Biol 2001 314 1041 1052 10.1006/jmbi.2000.5197 11743721

[B33] Tatusov RL Natale DA Garkavtsev IV Tatusova TA Shankavaram UT Rao BS Kiryutin B Galperin MY Fedorova ND Koonin EV The COG database: new developments in phylogenetic classification of proteins from complete genomes Nucleic Acids Res 2001 29 22 28 10.1093/nar/29.1.22 11125040 PMC29819

[B34] Dessimoz C Cannarozzi GM Gil M Margadant D Roth A Schneider A Gonnet GH OMA, A Comprehensive, Automated Project for the Identification of Orthologs from Complete Genome Data: Introduction and First Achievements 2005 LNCS 3678: Comparative Genomics: RECOMB 2005 International Workshop, RCG Dublin, Ireland

[B35] Demuth JP De Bie T Stajich JE Cristianini N Hahn MW The evolution of mammalian gene families PLoS ONE 2006 1 e85 10.1371/journal.pone.0000085 17183716 PMC1762380

[B36] Burmester T Ebner B Weich B Hankeln T Cytoglobin: A Novel Globin Type Ubiquitously Expressed in Vertebrate Tissues Mol Biol Evol 2002 19 416 421 11919282 10.1093/oxfordjournals.molbev.a004096

[B37] Aguileta G Bielawski JP Yang Z Proposed standard nomenclature for the alpha- and beta-globin gene families Genes Genet Syst 2006 81 367 371 10.1266/ggs.81.367 17159299

[B38] Rodriguez-Ezpeleta N Brinkmann H Burger G Roger AJ Gray MW Philippe H Lang BF Toward resolving the eukaryotic tree: the phylogenetic positions of jakobids and cercozoans Curr Biol 2007 17 1420 1425 10.1016/j.cub.2007.07.036 17689961

[B39] Horner DS Lefkimmiatis K Reyes A Gissi C Saccone C Pesole G Phylogenetic analyses of complete mitochondrial genome sequences suggest a basal divergence of the enigmatic rodent Anomalurus BMC Evol Biol 2007 7 16 10.1186/1471-2148-7-16 17288612 PMC1802082

[B40] Janecka JE Miller W Pringle TH Wiens F Zitzmann A Helgen KM Springer MS Murphy WJ Molecular and genomic data identify the closest living relative of primates Science 2007 318 792 794 10.1126/science.1147555 17975064

[B41] Kriegs JO Churakov G Jurka J Brosius J Schmitz J Evolutionary history of 7SL RNA-derived SINEs in Supraprimates Trends Genet 2007 23 158 161 10.1016/j.tig.2007.02.002 17307271

[B42] Corneli PS Complete Mitochondrial Genomes and Eutherian Evolution J Mamm Evol 2002 9 281 305 10.1023/A:1023926013667

[B43] Shoshani J McKenna MC Higher taxonomic relationships among extant mammals based on morphology, with selected comparisons of results from molecular data Mol Phylogenet Evol 1998 9 572 584 10.1006/mpev.1998.0520 9668007

[B44] Pumo DE Finamore PS Franek WR Phillips CJ Tarzami S Balzarano D Complete mitochondrial genome of a neotropical fruit bat, Artibeus jamaicensis, and a new hypothesis of the relationships of bats to other eutherian mammals J Mol Evol 1998 47 709 717 10.1007/PL00006430 9847413

[B45] Nishihara H Hasegawa M Okada N Pegasoferae, an unexpected mammalian clade revealed by tracking ancient retroposon insertions Proc Natl Acad Sci USA 2006 103 9929 9934 10.1073/pnas.0603797103 16785431 PMC1479866

[B46] Wible JR Rougier GW Novacek MJ Asher RJ Cretaceous eutherians and Laurasian origin for placental mammals near the K/T boundary Nature 2007 447 1003 1006 10.1038/nature05854 17581585

[B47] Mallet J Hybridization as an invasion of the genome Trends Ecol Evol 2005 20 229 237 10.1016/j.tree.2005.02.010 16701374

[B48] Hiendleder S Lewalski H Wolf E Janke A Complete mitochondrial genomes of *Bos taurus *and *Bos indicus *cattle provide novel aspects for taxonomy and domestication and reveal extent of intraspecific variation Cytogenet Cell Genet 2008 120 150 156 10.1159/000118756 18467841

[B49] Arnason U Spilliaert R Palsdottir A Arnason A Molecular identification of hybrids between the two largest whale species, the blue whale (Balaenoptera musculus) and the fin whale (B. physalus) Hereditas 1991 115 183 189 1687408 10.1111/j.1601-5223.1991.tb03554.x

[B50] Roy MS Girman DG Taylor AC Wayne RK The use of museum specimens to reconstruct the genetic variability and relationships of extinct populations Experientia 1994 50 551 557 10.1007/BF01921724 8020615

[B51] Ebersberger I Galgoczy P Taudien S Taenzer S Platzer M von Haeseler A Mapping human genetic ancestry Mol Biol Evol 2007 24 2266 2276 10.1093/molbev/msm156 17660505

[B52] Barton NH Evolutionary biology: how did the human species form? Curr Biol 2006 1616 647 650 10.1016/j.cub.2006.07.032 16920616

[B53] Patterson N Richter DJ Gnerre S Lander ES Reich D Genetic evidence for complex speciation of humans and chimpanzees Nature 2006 441 1103 1108 10.1038/nature04789 16710306

[B54] Osada N Wu CI Inferring the mode of speciation from genomic data: a study of the great apes Genetics 2005 169 259 564 10.1534/genetics.104.029231 15677748 PMC1448895

[B55] Nolte AW Freyhof J Stemshorn KC Tautz D An invasive lineage of sculpins, Cottus sp. (Pisces, Teleostei) in the Rhine with new habitat adaptations has originated from hybridization between old phylogeographic groups Proc Biol Sci 2005 272 2379 2387 10.1098/rspb.2005.3231 16243698 PMC1559961

[B56] Bininda-Emonds OR Cardillo M Jones KE MacPhee RD Beck RM Grenyer R Price SA Vos RA Gittleman JL Purvis A The delayed rise of present-day mammals Nature 2007 446 507 512 10.1038/nature05634 17392779

[B57] Boore JL The use of genome-level characters for phylogenetic reconstruction Trends Ecol Evol 2006 21 439 446 10.1016/j.tree.2006.05.009 16762445

[B58] Altschul SF Gish W Miller W Myers EW Lipman DJ Basic local alignment search tool J Mol Biol 1990 215 403 410 2231712 10.1016/S0022-2836(05)80360-2

[B59] Edgar RC MUSCLE: multiple sequence alignment with high accuracy and high throughput Nucleic Acids Res 2004 32 1792 1797 10.1093/nar/gkh340 15034147 PMC390337

[B60] Yang Z Roberts D On the use of nucleic acid sequences to infer early branchings in the tree of life Mol Biol Evol 1995 12 451 458 7739387 10.1093/oxfordjournals.molbev.a040220

[B61] Jobb G von Haeseler A Strimmer K TREEFINDER4: a powerful graphical analysis environment for molecular phylogenetics BMC Evol Biol 2004 4 18 10.1186/1471-2148-4-18 15222900 PMC459214

[B62] Strimmer K von Haeseler A Quartet puzzling: a quartet maximum likelihood method for reconstructing tree topologies Mol Biol Evol 1996 13 964 969

[B63] Guindon S Gascuel S A simple, fast and accurate algorithm to estimate large phylogenies by maximum likelihood Syst Biol 2003 52 696 704 10.1080/10635150390235520 14530136

[B64] Lanave C Preparata G Saccone C Serio G A new method for calculating evolutionary substitution rates J Mol Evol 1984 20 86 93 10.1007/BF02101990 6429346

[B65] Yang Z Maximum likelihood phylogenetic estimation from DNA sequences with variable rates over sites: approximate methods J Mol Evol 1994 39 306 314 10.1007/BF00160154 7932792

[B66] Whelan S Goldman N A general empirical model of protein evolution derived from multiple protein families using a maximum likelihood approach Mol Biol Evol 2001 18 691 699 11319253 10.1093/oxfordjournals.molbev.a003851

[B67] Shimodaira H Hasegawa M Multiple comparisons of log-likelihoods with applications to phylogenetic inference Mol Biol Evol 1999 16 1114 1116

[B68] Huelsenbeck JP Ronquist F MrBayes: Bayesian inference of phylogeny Bioinformatics 2001 17 754 755 10.1093/bioinformatics/17.8.754 11524383

[B69] Sanderson MJ R8s: inferring absolute rates of molecular evolution and divergence times in the absence of a molecular clock Bioinformatics 2002 19 301 302 10.1093/bioinformatics/19.2.301 12538260

[B70] Benton MJ Donoghue PC Paleontological evidence to date the tree of life Mol Biol Evol 2007 24 26 53 10.1093/molbev/msl150 17047029

